# The advanced lung cancer inflammation index is a prognostic factor for gastrointestinal cancer patients undergoing surgery: a systematic review and meta-analysis

**DOI:** 10.1186/s12957-023-02972-4

**Published:** 2023-03-06

**Authors:** Xu-Rui Liu, Lian-Lian Wang, Bin Zhang, Xiao-Yu Liu, Zi-Wei Li, Bing Kang, Chao Yuan, Zheng-Qiang Wei, Dong Peng

**Affiliations:** 1 Department of Gastrointestinal Surgery, The First Affiliated Hospital of Chongqing Medical University, Chongqing, 400016 China; 2 Department of Clinical Nutrition, the First Affiliated Hospital of Chongqing Medical University, Chongqing, 400016 China

**Keywords:** Advanced lung cancer inflammation index, Gastrointestinal cancer, Colorectal cancer, Gastric cancer, Surgery, Prognosis

## Abstract

**Background:**

The advanced lung cancer inflammation index (ALI) is a comprehensive assessment indicator that can reflect inflammation and nutrition conditions. However, there are some controversies about whether ALI is an independent prognostic factor for gastrointestinal cancer patients undergoing surgical resection. Thus, we aimed to clarify its prognostic value and explore the potential mechanisms.

**Methods:**

Four databases including PubMed, Embase, the Cochrane Library, and CNKI were used for searching eligible studies from inception to June 28, 2022. All gastrointestinal cancers, including colorectal cancer (CRC), gastric cancer (GC), esophageal cancer (EC), liver cancer, cholangiocarcinoma, and pancreatic cancer were enrolled for analysis. We focused on prognosis most in the current meta-analysis. Survival indicators, including overall survival (OS), disease-free survival (DFS), and cancer-special survival (CSS) were compared between the high ALI group and the low ALI group. The Preferred Reporting Items for Systematic Reviews and Meta-Analyses (PRISMA) checklist was submitted as a supplementary document.

**Results:**

We finally included fourteen studies involving 5091 patients in this meta-analysis. After pooling the hazard ratios (HRs) and 95% confidence intervals (CIs), ALI was found to be an independent prognostic factor for both OS (HR = 2.09, I^2^ = 92%, 95% CI = 1.53 to 2.85, *P* < 0.01), DFS (HR = 1.48, I^2^ = 83%, 95% CI = 1.18 to 1.87, *P* < 0.01), and CSS (HR = 1.28, I^2^ = 1%, 95% CI = 1.02 to 1.60, P = 0.03) in gastrointestinal cancer. After subgroup analysis, we found that ALI was still closely related to OS for CRC (HR = 2.26, I^2^ = 93%, 95% CI = 1.53 to 3.32, *P* < 0.01) and GC (HR = 1.51, I^2^ = 40%, 95% CI = 1.13 to 2.04, *P* = 0.006) patients. As for DFS, ALI also has a predictive value on the prognosis of CRC (HR = 1.54, I^2^ = 85%, 95% CI = 1.14 to 2.07, *P* = 0.005) and GC (HR = 1.37, I^2^ = 0%, 95% CI = 1.09 to 1.73, *P* = 0.007) patients.

**Conclusion:**

ALI affected gastrointestinal cancer patients in terms of OS, DFS, and CSS. Meanwhile, ALI was a prognostic factor both for CRC and GC patients after subgroup analysis. Patients with low ALI had poorer prognoses. We recommended that surgeons should perform aggressive interventions in patients with low ALI before the operation.

**Supplementary Information:**

The online version contains supplementary material available at 10.1186/s12957-023-02972-4.

## Introduction

Gastrointestinal cancer is common cancer that includes colorectal cancer (CRC) and gastric cancer (GC), esophageal cancer (EC), liver cancer, cholangiocarcinoma, and pancreatic cancer. According to the Global Cancer Epidemiological Statistics published in 2018, CRC was the fourth most common cancer and the second leading cause of cancer-related death, and GC was the third leading cause of cancer-related death [1]. Despite the continuous improvement of the economy and techniques, radical resection remains the main strategy for the treatment of gastrointestinal cancer [2–5]. However, the mortality of patients after surgery is still high, with a 5-year survival rate of nearly 50% [6–8]. Some studies have demonstrated that patients with low body mass index (BMI), low albumin (Alb) levels, and high inflammatory conditions have a higher risk of postoperative complications and poor survival [9–12]. More sensitive prognostic indicators are needed to instruct doctors to take measures in advance and to improve the survival and quality of life of gastrointestinal cancer patients.

The advanced lung cancer inflammation index (ALI) is a new marker. It is calculated by the patient's BMI, serum Alb level, and neutrophil-to-lymphocyte ratio (NLR) (ALI = BMI × ALB/NLR) [13]. Some studies have revealed that BMI has a prognostic value for malignant diseases [14, 15]. Lee J et al. performed a 16-study meta-analysis and revealed that overweight CRC patients had worse survival [15]. Alb is synthesized in the liver and is the main component of total serum protein in the body, which mainly reflects nutrition status [16]. Gonzalez-Trejo S reported that serum albumin was a prognostic factor for CRC [17]. NLR is the ratio of neutrophils to lymphocytes. When the body is in an inflammatory state, neutrophils are elevated, and lymphocytes are decreased [18, 19]. Moreover, high levels of neutrophils can promote tumor progression and inhibit the antitumor effects of lymphocytes [20]. Thus, the NLR was established as an inflammation indicator and could be considered a balance between pro-tumor status and antitumor status. Therefore, ALI could reflect the inflammation and nutrition state. Currently, some studies have reported that ALI can predict the survival of many cancers, including CRC [21–27], GC [28, 29], EC [30, 31], liver cancer [32], cholangiocarcinoma [33], pancreatic cancer [34, 35], and B-cell lymphoma [36].

Many studies have discussed the relationship between ALI and gastrointestinal cancer. Pian G, Yin CZ, and Barth DA et al. demonstrated that there was no significant difference in ALI between patients receiving CRC surgery or GC surgery [22, 28, 34]. Others thought that a low ALI level would lead to a poor prognosis for gastrointestinal cancer patients [21, 23–27, 29–33]. Therefore, we aimed to explore the exact prognostic ability of ALI for patients with gastrointestinal cancer undergoing surgery.

## Methods

We conducted this current meta-analysis in accordance with the Preferred Reporting Items for Systematic Reviews and Meta-Analyses (PRISMA) statement [37]. The Preferred Reporting Items for Systematic Reviews and Meta-Analyses (PRISMA) checklist was submitted as a supplementary document. The registration ID of this meta-analysis on PROSPERO is CRD42022362548, and the link is https://www.crd.york.ac.uk/prospero/display_record.php?ID=CRD42022362548.

### Literature search

We searched studies in four databases including PubMed, Embase, the Cochrane Library, and CNKI on Dec 10, 2022. ALI was searched as “Advanced lung cancer inflammation index” OR “ALI” OR “BMI x ALB / NLR” OR “BMI x serum albumin / NLR” OR “body mass index x serum albumin / neutrophil-to lymphocyte”. As for gastrointestinal cancer, the search strategy was “gastrointestinal cancer” OR “gastrointestinal neoplasms” OR “colon cancer” OR “rectal cancer” OR “colorectal cancer” OR “rectum cancer” OR “colorectal neoplasm” OR “colon neoplasm” OR “rectal neoplasm” OR “rectum neoplasm” OR “colorectal carcinoma” OR “colon carcinoma” OR “rectum carcinoma” OR “rectal carcinoma” OR “CRC” OR “gastric cancer” OR “gastric carcinoma” OR “gastric neoplasms” OR “stomach cancer” OR “stomach carcinoma” OR “stomach neoplasms” OR “liver cancer” OR “hepatocellular carcinoma cancer” OR “esophageal cancer” OR “esophageal neoplasm” OR “esophagus cancer” OR “esophagus neoplasm” OR “esophageal squamous cell carcinoma” OR “cholangiocarcinoma” OR “extrahepatic cholangiocarcinoma” OR “gallbladder cancer” OR “gallbladder neoplasms” OR “bile duct cancer” OR “bile duct neoplasms” OR “pancreatic cancer” OR “pancreatic carcinoma”. The search scope was limited to titles and abstracts. Language and study design had no limitations.

### Inclusion and exclusion criteria

The inclusion criteria for our meta-analysis included: 1, Patients with gastrointestinal cancer (CRC, GC, EC, liver cancer, cholangiocarcinoma, or pancreatic cancer) who received radical or palliative intent surgery; 2, Patients were divided into the high ALI group and the low ALI group; and 3, Prognosis including OS, DFS, or CSS was reported (both effect value and survival curves were allowed). The exclusion criteria included: 1, Studies’ types were reviews, case reports, letters, conferences, comments, or preprint articles; and 2, Data was repeated or overlapped. When two studies had overlapped data, the study with a larger sample size would be included. The PICO framework was more intuitive and was shown in a supplementary document.

### Study selection

According to the search strategy, two authors searched studies based on the search strategy in four databases independently. Titles and abstracts would be scanned first. Then, the full text was screened based on the inclusion and exclusion criteria. If there was a disagreement on study inclusion, a group discussion would be held to solve the resolution.

### Data collection

Two authors collected studies’ characteristics and patients’ information independently, then the data were checked to reach a consistent. Characteristics of the studies included the first author, year, country, study period, sample size, the cut-off value of ALI, prognosis, included patients, follow-up, and Newcastle–Ottawa Scale (NOS) score. The prognosis included OS, DFS, and CSS. The baseline information included age, sex, carcinoembryonic antigen (CEA), carbohydrate antigen 19–9 (CA 19–9), preoperative anemia, chemotherapy, lymph node metastasis, vessel invasion, neural invasion, distant metastasis, histology, and postoperative complication.

### Quality assessment

Our meta-analysis assessed the study quality in accordance with the Newcastle–Ottawa Scale (NOS) according to comparison selection, comparability between groups, and the determination of results [38]. High-quality studies have scores higher than 8 points, and medium-quality studies have scores of 7 or 8 points.

### Statistical analysis

In this meta-analysis, we focused on the prognosis of gastrointestinal cancer patients. The hazard ratios (HRs) and 95% confidence intervals (CIs) of OS, DFS, or CSS were pooled for analysis. HRs from the multivariate analysis were preferred, without which univariate analysis would be replaced. I^2^ value and chi-squared test were used to evaluate statistical heterogeneity. According to the Cochrane handbook, the I^2^ < 30% was considered low heterogeneity, the I^2^ range from 30 to 60% was considered moderate heterogeneity, and the I^2^ > 60% was considered high heterogeneity. The fixed effects model was used when the I^2^ value ≤ 50%, and *p* < 0.05 was thought of as statistically significant. The random effects model was used when the I^2^ value > 50%, and *p* < 0.1 was thought of as statistically significant. Subgroup analysis was used to assess the risk of heterogeneity. As for sensitive analysis, each study was excluded at a time, and repeat meta-analyses were conducted. The funnel plot was used for assessing the publication bias. RevMan 5.3 (The Cochrane Collaboration, London, United Kingdom) was used for all data analysis.

## Results

### Study selection

**Fig. 1:**
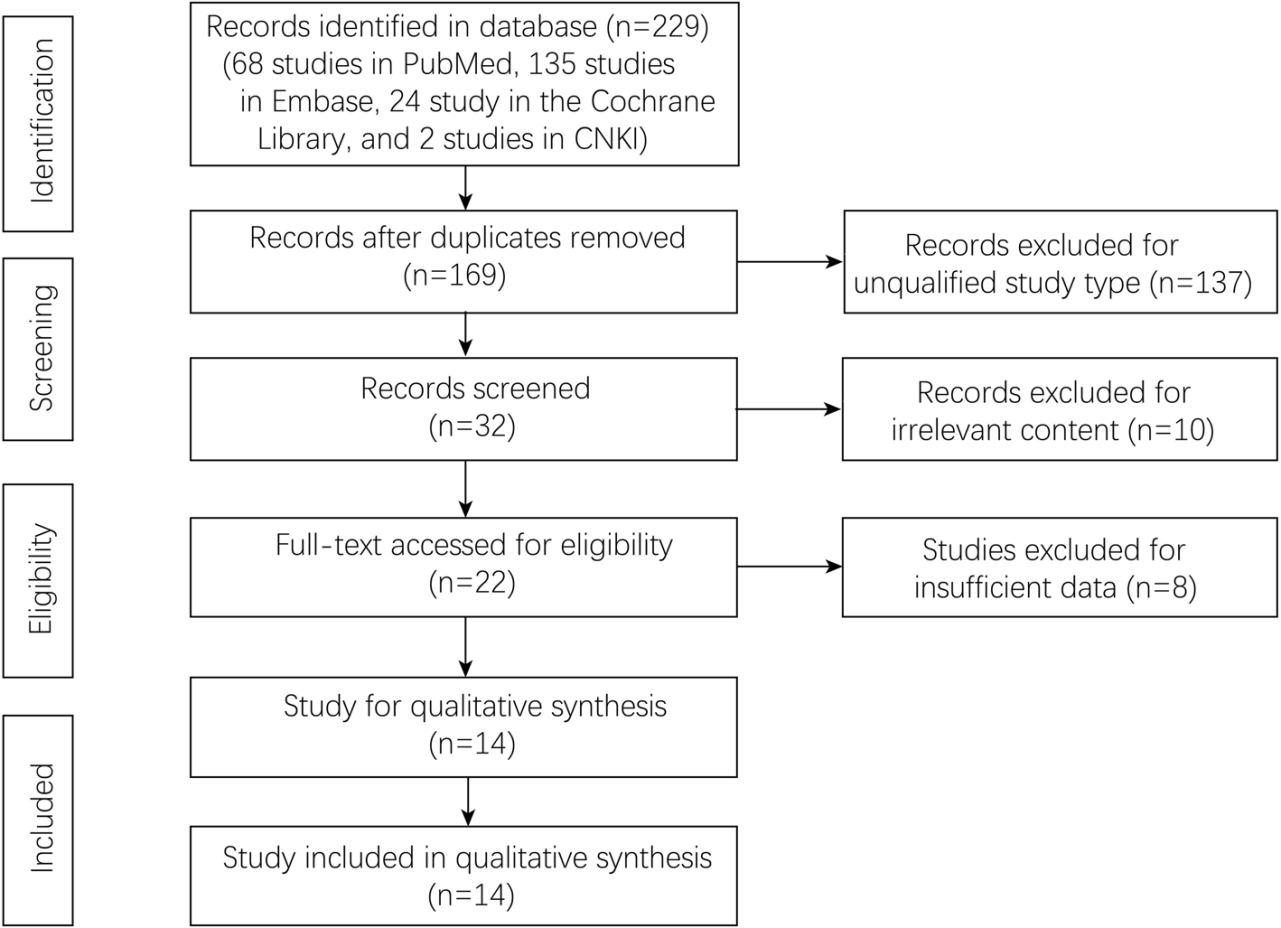
Flowchart of study selection

We searched 229 studies in four databases according to the search strategy (68 studies in PubMed, 135 studies in Embase, 24 studies in the Cochrane Library, and 2 studies in CNKI). After duplicates removing, the titles and abstracts were scanned for initial selection. Then, 22 studies were left for full-text assessment. Finally, 14 studies after qualitative synthesis with sufficient data were included (Fig. 1).

### Baseline characteristics of studies

Fourteen studies involving 5091 patients were included in the current meta-analysis. Studies were conducted in China, Japan, Korea, and Austria ranged from 2014 to 2022. The cut-off values of ALI ranged from 18.0 to 43.5 in eleven studies, and the value in another study was 70.4. Twelve studies reported OS, eight studies reported DFS/ progression-free survival (PFS)/ relapse-free survival (RFS), and two studies reported CSS. More information, including study period, sample size, cancer type of included patients, follow-up, and NOS scores are also shown in Table 1.

**Table 1:** Characteristics of the studies included in the meta-analysis

Author	Year	Country	Study date	Sample size			Cut-off value	Prognosis	Patients included	Follow-up (month)	NOS score
				High ALI	Low ALI	Total					
Horino T [21]	2021	Japan	2005–2019	532	281	813	NA	OS、RFS	CRC	NA	9
Pian G [22]	2020	Korea	2009–2018	32	100	132	70.4	OS、DFS	CRC patients with liver metastases	NA	6
Kusunoki K [23]	2020	Japan	2005–2011	224	74	298	NA	OS、DFS	CRC	36.8 (39.7 ± 29.0)^a^	8
Xie HL [24]	2020	China	2012–2014	423	239	662	manle31.6; female24.4	OS、PFS	CRC	63 (1–80)^b^	9
Shinutani M [25]	2019	Japan	2008–2016	92	67	159	28.9	OS	unresectable metastatic CRC patients underwent combination chemotherapy	21.6 (1.2–94.0)^b^	6
Chen C [26]	2022	China	2012–2016	130	179	309	25.71	OS	CRC	60 (1–98)^b^	8
Yin CZ [28]	2021	Japan	1992–2011	NA	NA	620	30.0	OS、DFS	GC	52.8 ± 39.9^c^	9
Zhang X [29]	2022	China	2010–2017	362	253	615	39.77	OS、DFS	GC	40 (25–64)^b^	9
Deng Y [27]	2022	China	2012–2016	205	236	441	36.3	OS、DFS	right-sided colon cancer	65 (3–110)^b^	8
Tan X [30]	2021	China	2013–2018	57	101	158	31.24	OS	EC	29–88^d^	7
Feng JF [31]	2014	China	2006–2008	173	120	293	18.0	CSS	EC	35–71^d^	8
Li Q [32]	2022	China	2017–2020	35	30	65	34.7	OS	advanced HCC	at least 24 months	7
Wu H [33]	2022	China	2016–2019	35	62	97	31.8	OS、DFS	cholangiocarcinoma	20 (3–70)^b^	7
Barth DA [34]	2020	Austria	2003–2015	NA	NA	429	43.5	CSS	PC	NA	9

*Abbreviations: CRC* colorectal cancer, *GC* gastric cancer, *EC* Esophageal cancer, *HCC* hepatocellular carcinoma, *PC* pancreatic cancer; NOS, Newcastle–Ottawa Scale, *OS* overall survival, *DFS* disease-free survival, *PFS* progression-free survival, *RFS* relapse-free survival, *CSS* cancer-specific survival

^a^median (mean ± standard deviation)

^b^median (ranges)

^c^mean ± standard deviation

^d^range

### Baseline characteristics of patients

Compared to the low ALI group, the high ALI group had a lower proportion of older patients (OR = 0.74, I^2^ = 0%, 95% CI = 0.64 to 0.85, *P* < 0.0001) and males (OR = 0.49, I^2^ = 92%, 95% CI = 0.25 to 0.98, *P* = 0.04), less preoperative anemia (OR = 0.53, I^2^ = 0%, 95% CI = 0.36 to 0.78, *P* = 0.001), chemotherapy (OR = 0.75, I^2^ = 0%, 95% CI = 0.61 to 0.92, *P* = 0.006), less distant metastasis (OR = 0.42, I^2^ = 52%, 95% CI = 0.26 to 0.66, *P* = 0.0002). No significant differences were found in CEA levels, CA 19–9, lymph node metastasis, vessel invasion, neural invasion, histology, and postoperative complication (*P* ≥ 0.05 in the fixed effects model or *P* ≥ 0.1 in the random effects model) (Table 2).

**Table 2:** Summary of characteristics between High ALI group and Low ALI group

Characteristics	Studies	Participants (High ALI/ Low ALI)	Odds Ratio (95% CI)	Model	Heterogeneity
Age					
Young	8	Reference	Reference	Reference	Reference
Old	8	2056/ 1426	0.74 [0.64, 0.85]; *P* < 0.0001	FE	I^2^ = 0%; *P* = 0.83
Sex					
Female	8	Reference	Reference	Reference	Reference
Male	8	1799/ 1238	0.49 [0.25, 0.98]; *P* = 0.04	RE	I^2^ = 92%; *P* < 0.00001
CEA					
≤ 5	6	Reference	Reference	Reference	Reference
> 5	6	1359/ 1014	0.74 [0.49, 1.11]; *P* = 0.15	RE	I^2^ = 78%; *P* = 0.0003
CA 19–9					
≤ 37	3	Reference	Reference	Reference	Reference
> 37	3	774/ 496	0.87 [0.65, 1.16]; *P* = 0.35	FE	I^2^ = 20%; *P* = 0.29
Preoperative anemia	2	242/ 215	0.53 [0.36, 0.78]; *P* = 0.001	FE	I^2^ = 0%; *P* = 0.96
Chemotherapy	5	878/ 832	0.75 [0.61, 0.92]; *P* = 0.006	FE	I^2^ = 0%; *P* = 0.47
Lymph node metastasis	3	936/ 535	1.00 [0.78, 1.28]; *P* = 0.98	FE	I^2^ = 37%; *P* = 0.20
Vessel invasion	4	950/ 612	0.78 [0.60, 1.02]; *P* = 0.07	FE	I^2^ = 0%; *P* = 0.75
Neural invasion	3	372/ 384	0.75 [0.51, 1.12]; *P* = 0.16	FE	I^2^ = 3%; *P* = 0.36
Distant metastasis	3	777/ 492	0.42 [0.26, 0.66]; *P* = 0.0002	RE	I^2^ = 52%; *P* = 0.13
Histology					
Well/ Moderate	7	Reference	Reference	Reference	Reference
Poor	7	1554/ 1080	0.16 [0.02, 1.46]; *P* = 0.10	RE	I^2^ = 99%; *P* < 0.00001
Postoperative complication	3	626/ 416	0.74 [0.36, 1.53]; *P* = 0.41	RE	I^2^ = 54%; *P* = 0.12

*Abbreviations: ALI* advanced lung cancer inflammation index, *CI* confidence intervals, *CEA* carcinoembryonic antigen, *CA 19–9* carbohydrate antigen 19–9

### Clinical impact of the preoperative ALI on the survival outcome

**Fig. 2:**
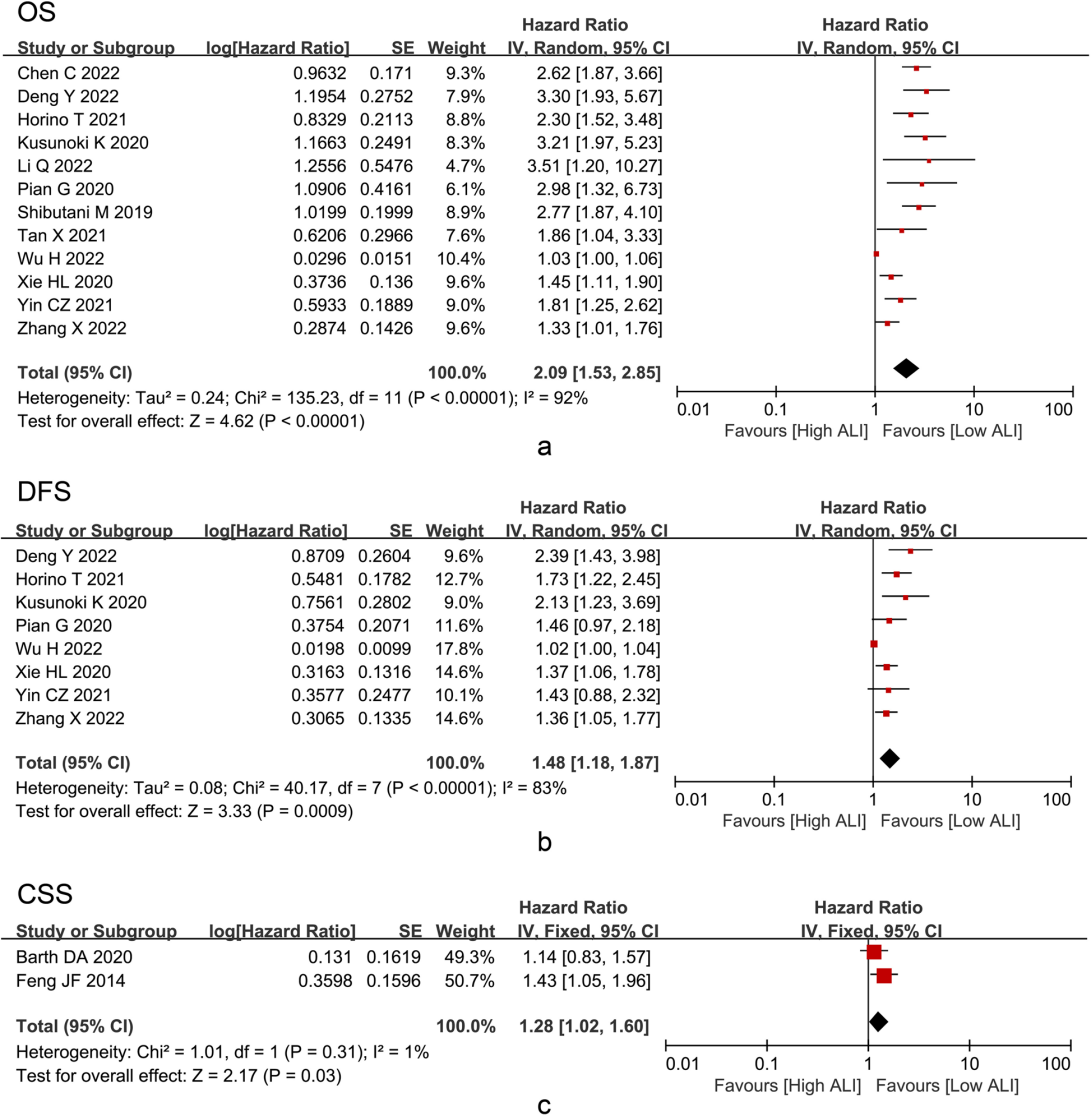
**a** OS, **b** DFS, and **c** CSS of the low ALI group and the high ALI group. Abbreviations: OS, overall survival; DFS, disease-free survival; CSS, cancer-specific survival; ALI, advanced lung cancer inflammation index

**Fig. 3:**
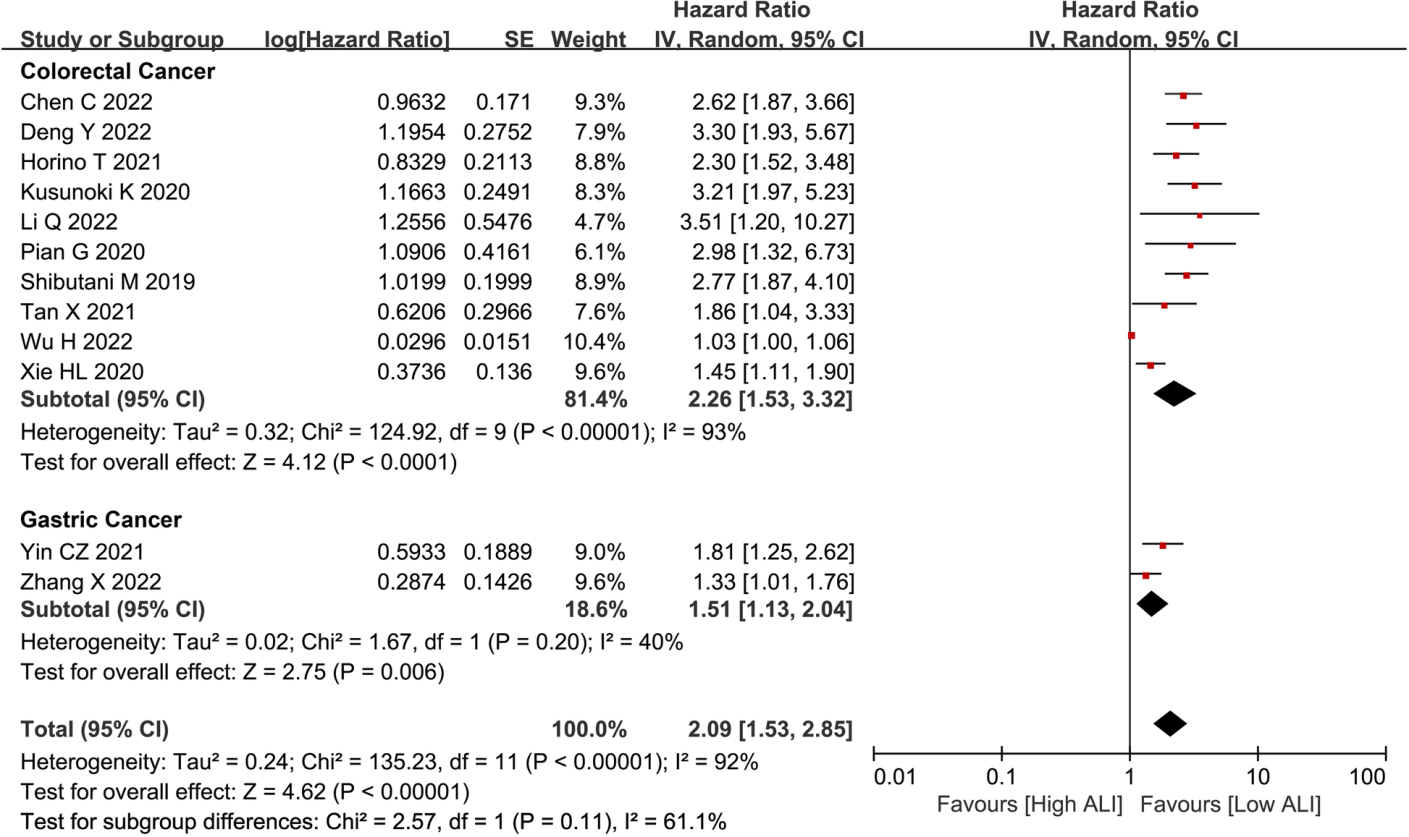
Subgroup analysis for OS. Abbreviations: OS, overall survival; ALI, advanced lung cancer inflammation index

**Fig. 4:**
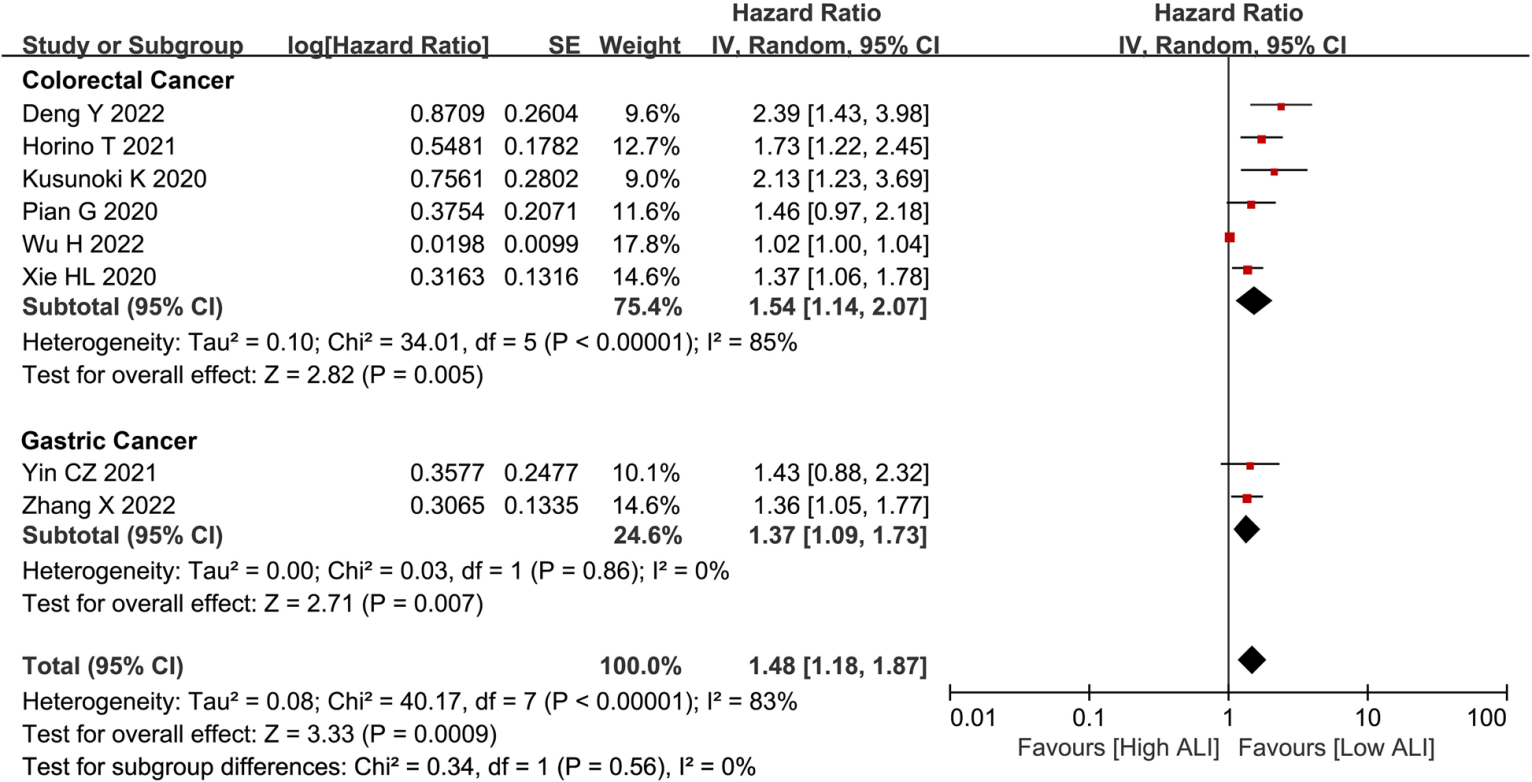
Subgroup analysis for DFS. Abbreviations: DFS, disease-free survival; ALI, advanced lung cancer inflammation index

After pooling the HRs and 95% CIs of OS from fourteen studies, ALI was a prognostic predictor for OS (HR = 2.09, I^2^ = 92%, 95% CI = 1.01 to 1.76, P < 0.01), DFS (HR = 1.48, I^2^ = 83%, 95% CI = 1.18 to 1.87, *P* < 0.01) and CSS (HR = 1.28, I^2^ = 1%, 95% CI = 1.02 to 1.60, P = 0.03) in gastrointestinal cancer patients after surgery (Fig. 2). Subgroup analysis was conducted for CRC patients and GC patients independently. As for OS, we found that there was still a close association between ALI and CRC (HR = 2.26, I^2^ = 93%, 95% CI = 1.53 to 3.32, *P* < 0.01) and GC (HR = 1.51, I^2^ = 40%, 95% CI = 1.13 to 2.04, *P* = 0.006) (Fig. 3). As for DFS, the prognostic value of ALI was also shown in CRC (HR = 1.54, I^2^ = 85%, 95% CI = 1.14 to 2.07, *P* = 0.005) and GC (HR = 1.37, I^2^ = 0%, 95% CI = 1.09 to 1.73, *P* = 0.007) (Fig. 4).

### Sensitivity analysis

**Fig. 5:**
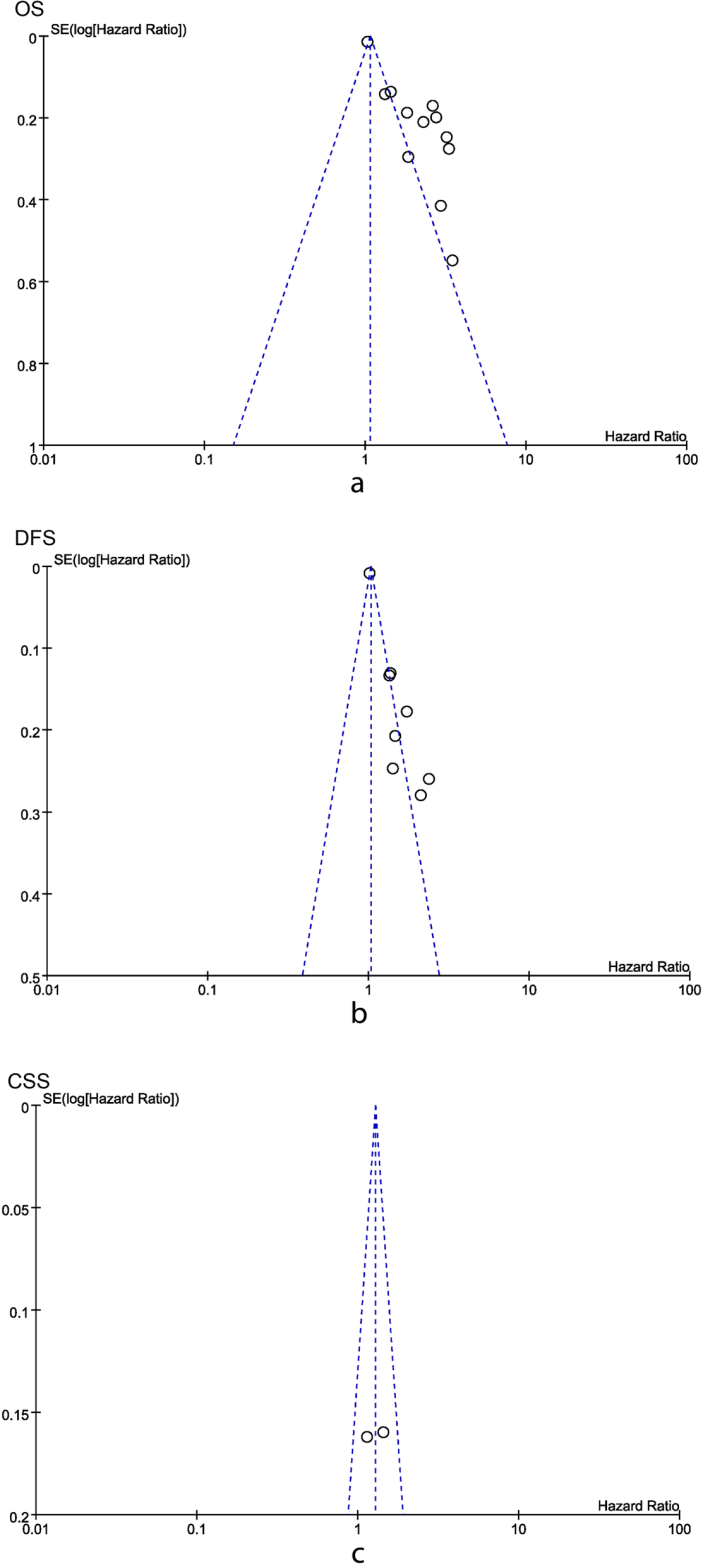
Funnel plots for **a** OS, **b** DFS, and **c** CSS

We excluded each study at a time for repeated analysis, and the exclusion of any one study did not significantly alter the results. The funnel plot was performed for assessing the publication bias of OS, DFS, and CSS (Fig. 5). Unfortunately, only a small publication bias was found in the CSS due to the symmetry of its funnel plot, which meant that the result of the CSS was reliable. The source of publication bias in OS and DFS might be that no published studies with a negative outcome or no correlation were found currently.

## Discussion

Our meta-analysis enrolled 5091 patients from fourteen studies. We found that a low ALI was an independent prognostic factor for OS, DFS, and CSS in gastrointestinal cancer patients. Further subgroup analysis reported that ALI had prognostic effects for both CRC and GC patients.

Gastrointestinal cancer is a malignant wasting disease that can be accompanied by obstruction or bleeding, which causes malnutrition [39]. Malnutrition was related to high incidences of poor survival and postoperative complications [40]. Therefore, some nutritional parameters including BMI [14, 15] and Alb levels [16, 17], have prognostic value for gastrointestinal cancer patients. ALI is a combined score that is calculated by BMI, Alb levels, and NLR. The prognostic value of ALI was first reported in non-small cell lung cancer [13]. It can reflect the nutrition status of the host [13].

Some studies have researched the relationship between ALI and gastrointestinal cancer. Most studies demonstrated that ALI could predict prognosis including OS and DFS for colorectal cancer patients [21, 23–25, 27]. Zhang X showed that preoperative ALI was an independent prognostic factor for GC patients undergoing curative gastrectomy [29]. ALI was also revealed to be a prognostic predictor for EC, liver cancer, and cholangiocarcinoma [30–33]. However, Pian G, Yin CZ, and Barth DA reported that there was no significant difference between ALI and DFS after gastrointestinal surgery [22, 28, 34]. Therefore, the extra relationship and potential mechanism need to be analyzed.

ALI not only predicts the prognosis of gastrointestinal cancer patients by reflecting the body's nutritional status but also the response to inflammatory conditions. It was reported that inflammation was an important process for the occurrence and development of gastrointestinal cancer [41]. When inflammatory factors and inflammatory cells were activated, new lymphatic vessels and blood vessels formed, which created a microenvironment conducive to the growth and differentiation of tumor cells. Tumors can also disrupt immune cell function, and then, tumor cells can more easily invade and metastasize. Many inflammatory indicators, including the albumin-to-globulin ratio (AGR), NLR, and systemic immune-inflammation index, are independent prognostic factors for gastrointestinal cancer [42–47]. ALI, an inflammatory indicator, might also have use for gastrointestinal cancer patients.

Current studies are controversial regarding the prognostic predictive value of ALI in patients with gastrointestinal cancers. Our meta-analysis was the first study to summarize the outcomes and mainly solved the inconsistency of ALI in DFS. Moreover, the included studies were relatively new, with the earliest one published in 2019. Then, we included all gastrointestinal cancer studies for analysis. In addition, almost all the included studies were published in Asia, which means that the results of our study are reliable for Asian individuals. However, the results could not be extrapolated worldwide because of the differences in metabolic profiles between different nations.

However, there were some other limitations in this meta-analysis. First, we only included fourteen retrospective studies without any randomized controlled trials or cohort studies. Second, the cutoff value of studies was not consistent, which might lead to error. Third, the potential association between ALI and curative or palliative surgery needs to be explored further.

In conclusion, gastrointestinal cancer patients with a low ALI had a higher risk of poor prognosis after surgery. ALI was an independent prognostic factor for both OS and DFS. Doctors need to pay more attention to patients with low ALI to improve their prognosis.

## Supplementary Information

**Additional file 1.** PRISMA 2020 Checklist.

**Additional file 2.** PICO Framework.

**Additional file 3.** Search Strategy.

**Additional file 4.** PRISMA flow chart.

**Additional file 5: Table S1.** Results of quality assessment using the Newcastle-Ottawa Scale for cohort studies.

**Additional file 6: Table S2.** 

## Data Availability

All the data used in this study can be obtained from the original articles.

## Abbreviations

CRC: Colorectal cancer
GC: Gastric cancer
ALI: Advanced lung cancer inflammation index
OS: Overall survival
DFS: Disease-free survival
PFS: Progression-free survival
RFS: Relapse-free survival
HRs: Hazard ratios
CIs: Confidence intervals
BMI: Body mass index
Alb: Albumin
TNM: Tumor node metastasis
NLR: Neutrophil-to-lymphocyte ratio
CEA: Carcinoembryonic antigen
NOS: Newcastle–Ottawa Scale
PRISMA: Preferred Reporting Items for Systematic Reviews and Meta-Analyses

## References

[1] Bray F, Ferlay J, Soerjomataram I (2018). Global cancer statistics 2018: GLOBOCAN estimates of incidence and mortality worldwide for 36 cancers in 185 countries. CA Cancer J Clin.

[2] Nordlinger B, Sorbye H, Glimelius B (2013). Perioperative FOLFOX4 chemotherapy and surgery versus surgery alone for resectable liver metastases from colorectal cancer (EORTC 40983): long-term results of a randomised, controlled, phase 3 trial. Lancet Oncol.

[3] Peng D, Cheng YX, Cheng Y (2021). Improved Overall Survival of Colorectal Cancer under Multidisciplinary Team: A Meta-Analysis. Biomed Res Int.

[4] Peng D, Liu XY, Cheng YX (2021). Improvement of Diabetes Mellitus After Colorectal Cancer Surgery: A Retrospective Study of Predictive Factors for Type 2 Diabetes Mellitus Remission and Overall Survival. Front Oncol..

[5] Peng D, Cheng YX, Tao W (2020). Onco-metabolic surgery: a combined approach to gastric cancer and hypertension. Cancer Manag Res.

[6] Cheng YX, Tao W, Kang B (2022). Impact of preoperative type 2 diabetes mellitus on the outcomes of gastric cancer patients following gastrectomy: a propensity score matching analysis. Front Surg..

[7] Smyth EC, Nilsson M, Grabsch HI (2020). Gastric cancer. Lancet.

[8] Tarantino I, Warschkow R, Worni M (2015). Prognostic relevance of palliative primary tumor removal in 37,793 metastatic colorectal cancer patients: a population-based, propensity score-adjusted trend analysis. Ann Surg.

[9] Zhang L, Shi FY, Qin Q (2022). Relationship between preoperative inflammatory indexes and prognosis of patients with rectal cancer and establishment of prognostic nomogram prediction model. Zhonghua Zhong Liu Za Zhi..

[10] Doleman B, Mills KT, Lim S (2016). Body mass index and colorectal cancer prognosis: a systematic review and meta-analysis. Tech Coloproctol.

[11] Almasaudi AS, Dolan RD, Edwards CA, McMillan DC (2020). Hypoalbuminemia reflects nutritional risk, body composition and systemic inflammation and is independently associated with survival in patients with colorectal cancer. Cancers (Basel).

[12] Miyamoto Y, Baba Y, Sakamoto Y (2015). Sarcopenia is a negative prognostic factor after curative resection of colorectal cancer. Ann Surg Oncol.

[13] Jafri SH, Shi R, Mills G (2013). Advance lung cancer inflammation index (ALI) at diagnosis is a prognostic marker in patients with metastatic non-small cell lung cancer (NSCLC): a retrospective review. BMC Cancer.

[14] Renfro LA, Loupakis F, Adams RA (2016). Body Mass index is prognostic in metastatic colorectal cancer: pooled analysis of patients from first-line clinical trials in the ARCAD database. J Clin Oncol.

[15] Lee J, Meyerhardt JA, Giovannucci E, Jeon JY (2015). Association between body mass index and prognosis of colorectal cancer: a meta-analysis of prospective cohort studies. PLoS One..

[16] Tan CS, Read JA, Phan VH (2015). The relationship between nutritional status, inflammatory markers and survival in patients with advanced cancer: a prospective cohort study. Support Care Cancer.

[17] González-Trejo S, Carrillo JF, Carmona-Herrera DD (2017). Baseline serum albumin and other common clinical markers are prognostic factors in colorectal carcinoma: A retrospective cohort study. Medicine (Baltimore).

[18] Forget P, Dinant V, De Kock M (2015). Is the Neutrophil-to-Lymphocyte Ratio more correlated than C-reactive protein with postoperative complications after major abdominal surgery?. PeerJ.

[19] Kim H, Jung HI, Kwon SH (2019). Preoperative neutrophil-lymphocyte ratio and CEA is associated with poor prognosis in patients with synchronous colorectal cancer liver metastasis. Ann Surg Treat Res.

[20] Proctor MJ, McMillan DC, Morrison DS (2012). A derived neutrophil to lymphocyte ratio predicts survival in patients with cancer. Br J Cancer.

[21] Horino T, Tokunaga R, Miyamoto Y (2021). The advanced lung cancer inflammation index is a novel independent prognosticator in colorectal cancer patients after curative resection. Ann Gastroenterol Surg.

[22] Pian G, Hong SY, Oh SY (2022). Prognostic value of advanced lung cancer inflammation index in patients with colorectal cancer liver metastases undergoing surgery. Tumori.

[23] Kusunoki K, Toiyama Y, Okugawa Y (2020). Advanced lung cancer inflammation index predicts outcomes of patients with colorectal cancer after surgical resection. Dis Colon Rectum.

[24] Xie H, Huang S, Yuan G (2020). The advanced lung cancer inflammation index predicts short and long-term outcomes in patients with colorectal cancer following surgical resection: a retrospective study. PeerJ.

[25] Shibutani M, Maeda K, Nagahara H (2019). The prognostic significance of the advanced lung cancer inflammation index in patients with unresectable metastatic colorectal cancer: a retrospective study. BMC Cancer.

[26] Chen C, Mao S, Li M (2022). Correlation between inflammation index and colorectal cancer prognosis in advanced preoperative lung cancer. Chin J Can Prev Treatment.

[27] Deng Y, Sun Y, Lin Y, Huang Y, Chi P (2022). Clinical implication of the advanced lung cancer inflammation index in patients with right-sided colon cancer after complete mesocolic excision: a propensity score-matched analysis. World J Surg Oncol..

[28] Yin C, Toiyama Y, Okugawa Y (2021). Clinical significance of advanced lung cancer inflammation index, a nutritional and inflammation index, in gastric cancer patients after surgical resection: a propensity score matching analysis. Clin Nutr.

[29] Zhang X, Wang D, Sun T (2022). Advanced lung cancer inflammation index (ALI) predicts prognosis of patients with gastric cancer after surgical resection. BMC Cancer.

[30] Tan X, Peng H, Gu P, Chen M, Wang Y (2021). Prognostic significance of the l3 skeletal muscle index and advanced lung cancer inflammation index in elderly patients with esophageal cancer. Cancer Manag Res..

[31] Feng JF, Huang Y, Chen QX (2014). A new inflammation index is useful for patients with esophageal squamous cell carcinoma. Onco Targets Ther..

[32] Li Q, Ma F, Tsilimigras DI, Aberg F, Wang JF (2022). The value of the Advanced Lung Cancer Inflammation Index (ALI) in assessing the prognosis of patients with hepatocellular carcinoma treated with camrelizumab: a retrospective cohort study. Annals of Translational Medicine.

[33] Wu H, Ding F, Lin M (2022). Use of the advanced lung cancer inflammation index as a prognostic indicator for patients with cholangiocarcinoma. Front Surg.

[34] Barth DA, Brenner C, Riedl JM (2020). External validation of the prognostic relevance of the advanced lung cancer inflammation index (ALI) in pancreatic cancer patients. Cancer Med.

[35] Topkan E, Mertsoylu H, Ozdemir Y (2019). Prognostic usefulness of advanced lung cancer inflammation index in locally-advanced pancreatic carcinoma patients treated with radical chemoradiotherapy. Cancer Manag Res.

[36] Hu BL, Chen LH, Xu JQ (2018). The prognostic value of lower pretreatment advanced lung cancer inflammation index (ALI) in B cell lymphoma. Zhonghua Xue Ye Xue Za Zhi..

[37] Page MJ, McKenzie JE, Bossuyt PM (2021). The PRISMA 2020 statement: An updated guideline for reporting systematic reviews. J Clin Epidemiol.

[38] Stang A (2010). Critical evaluation of the Newcastle-Ottawa scale for the assessment of the quality of nonrandomized studies in meta-analyses. Eur J Epidemiol.

[39] Alifano M, Mansuet-Lupo A, Lococo F (2014). Systemic inflammation, nutritional status and tumor immune microenvironment determine outcome of resected non-small cell lung cancer. PLoS One..

[40] Hall JC (2006). Nutritional assessment of surgery patients. J Am Coll Surg.

[41] Nasr R, Salim Hammoud M, Nassar F (2018). Inflammatory Markers and MicroRNAs: The Backstage Actors Influencing Prognosis in Colorectal Cancer Patients. Int J Mol Sci.

[42] Moore MM, Chua W, Charles KA, Clarke SJ (2010). Inflammation and cancer: causes and consequences. Clin Pharmacol Ther.

[43] Xie QK, Chen P, Hu WM (2018). The systemic immune-inflammation index is an independent predictor of survival for metastatic colorectal cancer and its association with the lymphocytic response to the tumor. J Transl Med.

[44] Song Y, Yang Y, Gao P (2017). The preoperative neutrophil to lymphocyte ratio is a superior indicator of prognosis compared with other inflammatory biomarkers in resectable colorectal cancer. BMC Cancer.

[45] Pedrazzani C, Mantovani G, Fernandes E (2017). Assessment of neutrophil-to-lymphocyte ratio, platelet-to-lymphocyte ratio and platelet count as predictors of long-term outcome after R0 resection for colorectal cancer. Sci Rep.

[46] Fujikawa H, Toiyama Y, Inoue Y (2017). Prognostic Impact of Preoperative Albumin-to-Globulin Ratio in Patients with Colon Cancer Undergoing Surgery with Curative Intent. Anticancer Res.

[47] Shimura T, Toiyama Y, Saigusa S (2017). Inflammation-based prognostic scores as indicators to select candidates for primary site resection followed by multimodal therapy among colorectal cancer patients with multiple metastases. Int J Clin Oncol.

