# Supplementary material for: The advanced lung cancer inflammation index is a prognostic factor for gastrointestinal cancer patients undergoing surgery: a systematic review and meta-analysis
Source: World J Surg Oncol. 2023 Mar 6;21:81. doi: 10.1186/s12957-023-02972-4 (PMC9987069; doi:10.1186/s12957-023-02972-4)
Supplement: Supplementary file 1 — Additional file 1. PRISMA 2020 Checklist. [file 12957_2023_2972_MOESM1_ESM.docx]

Question: Is the advanced lung cancer inflammation index (ALI) a prognostic factor for gastrointestinal cancer patients undergoing surgery?

| Framework item |  |
| --- | --- |
| Patient/ Population | Patients with gastrointestinal cancer including colorectal cancer, gastric cancer, esophageal cancer, liver cancer, cholangiocarcinoma, and pancreatic cancer received radical or palliative surgery. |
| Intervention | Patients with low preoperative ALI level |
| Comparison | Patients with high preoperative ALI level |
| Outcome | Survival rates, death rates, cancer recurrence rate, and cancer progression |
