# Supplementary material for: The advanced lung cancer inflammation index is a prognostic factor for gastrointestinal cancer patients undergoing surgery: a systematic review and meta-analysis
Source: World J Surg Oncol. 2023 Mar 6;21:81. doi: 10.1186/s12957-023-02972-4 (PMC9987069; doi:10.1186/s12957-023-02972-4)
Supplement: Supplementary file 3 — Additional file 3. Search Strategy. [file 12957_2023_2972_MOESM3_ESM.docx]

Search Strategy

PubMed

#1

("advanced lung cancer inflammation index"[Title/Abstract] OR "ALI"[Title/Abstract] OR (("BMI"[All Fields] AND "x"[All Fields]) AND "alb nlr"[Title/Abstract]) OR (("BMI"[All Fields] AND "x"[All Fields] AND ("serum"[MeSH Terms] OR "serum"[All Fields] OR "serums"[All Fields] OR "serum s"[All Fields] OR "serumal"[All Fields])) AND "albumin nlr"[Title/Abstract]) OR ((("body mass index"[MeSH Terms] OR ("body"[All Fields] AND "mass"[All Fields] AND "index"[All Fields]) OR "body mass index"[All Fields]) AND "x"[All Fields]) AND "serum albumin neutrophil to lymphocyte"[Title/Abstract])) AND ("gastrointestinal cancer"[Title/Abstract] OR "gastrointestinal neoplasms"[Title/Abstract] OR "colon cancer"[Title/Abstract] OR "rectal cancer"[Title/Abstract] OR "colorectal cancer"[Title/Abstract] OR "rectum cancer"[Title/Abstract] OR "colorectal neoplasm"[Title/Abstract] OR "colon neoplasm"[Title/Abstract] OR "rectal neoplasm"[Title/Abstract] OR "rectum neoplasm"[Title/Abstract] OR "colorectal carcinoma"[Title/Abstract] OR "colon carcinoma"[Title/Abstract] OR "rectum carcinoma"[Title/Abstract] OR "rectal carcinoma"[Title/Abstract] OR "CRC"[Title/Abstract] OR "gastric cancer"[Title/Abstract] OR "gastric carcinoma"[Title/Abstract] OR "gastric neoplasms"[Title/Abstract] OR "stomach cancer"[Title/Abstract] OR "stomach carcinoma"[Title/Abstract] OR "stomach neoplasms"[Title/Abstract] OR “liver cancer” [Title/Abstract] OR “hepatocellular carcinoma cancer” [Title/Abstract] OR “esophageal cancer” [Title/Abstract] OR “esophageal neoplasm” [Title/Abstract] OR “esophagus cancer” [Title/Abstract] OR “esophagus neoplasm” [Title/Abstract] OR “esophageal squamous cell carcinoma” [Title/Abstract] OR “cholangiocarcinoma” [Title/Abstract] OR “extrahepatic cholangiocarcinoma” [Title/Abstract] OR “gallbladder cancer” [Title/Abstract] OR “gallbladder neoplasms” [Title/Abstract] OR “bile duct cancer” [Title/Abstract] OR “bile duct neoplasms” [Title/Abstract] OR “pancreatic cancer” [Title/Abstract] OR “pancreatic carcinoma” [Title/Abstract])

Embase

#2

('advanced lung cancer inflammation index':ti,ab,kw OR 'ali':ti,ab,kw OR 'bmi x alb / nlr':ti,ab,kw OR 'bmi x serum albumin / nlr':ti,ab,kw OR 'body mass index x serum albumin / neutrophil-to lymphocyte':ti,ab,kw) AND ('gastrointestinal cancer':ti,ab,kw OR 'gastrointestinal neoplasms':ti,ab,kw OR 'colon cancer':ti,ab,kw OR 'rectal cancer':ti,ab,kw OR 'colorectal cancer':ti,ab,kw OR 'rectum cancer':ti,ab,kw OR 'colorectal neoplasm':ti,ab,kw OR 'colon neoplasm':ti,ab,kw OR 'rectal neoplasm':ti,ab,kw OR 'rectum neoplasm':ti,ab,kw OR 'colorectal carcinoma':ti,ab,kw OR 'colon carcinoma':ti,ab,kw OR 'rectum carcinoma':ti,ab,kw OR 'rectal carcinoma':ti,ab,kw OR 'crc':ti,ab,kw OR 'gastric cancer':ti,ab,kw OR 'gastric carcinoma':ti,ab,kw OR 'gastric neoplasms':ti,ab,kw OR 'stomach cancer':ti,ab,kw OR 'stomach carcinoma':ti,ab,kw OR 'stomach neoplasms':ti,ab,kw OR ‘liver cancer’ :ti,ab,kw OR ‘hepatocellular carcinoma cancer’ :ti,ab,kw OR ‘esophageal cancer’ :ti,ab,kw OR ‘esophageal neoplasm’ :ti,ab,kw OR ‘esophagus cancer’ :ti,ab,kw OR ‘esophagus neoplasm’ :ti,ab,kw OR ‘esophageal squamous cell carcinoma’ :ti,ab,kw OR ‘cholangiocarcinoma’ :ti,ab,kw OR ‘extrahepatic cholangiocarcinoma’ :ti,ab,kw OR ‘gallbladder cancer’ :ti,ab,kw OR ‘gallbladder neoplasms’ :ti,ab,kw OR ‘bile duct cancer’ :ti,ab,kw OR ‘bile duct neoplasms’ :ti,ab,kw OR ‘pancreatic cancer’ :ti,ab,kw OR ‘pancreatic carcinoma’ :ti,ab,kw)

Cochrane Library

#3

(“Advanced lung cancer inflammation index” OR “ALI”):ti,ab,kw AND (“Gastrointestinal cancer” OR “Gastrointestinal Neoplasms” OR “Colon cancer” OR “rectal cancer” OR “colorectal cancer” OR “rectum cancer” OR “colorectal neoplasm” OR “colon neoplasm” OR “rectal neoplasm” OR “rectum neoplasm” OR “colorectal carcinoma” OR “colon carcinoma” OR “rectum carcinoma” OR “rectal carcinoma” OR “CRC” OR “gastric cancer” OR “gastric carcinoma” OR “gastric neoplasms” OR “stomach cancer” OR “stomach carcinoma” OR “stomach neoplasms” OR “liver cancer” OR “hepatocellular carcinoma cancer” OR “esophageal cancer” OR “esophageal neoplasm” OR “esophagus cancer” OR “esophagus neoplasm” OR “esophageal squamous cell carcinoma” OR “cholangiocarcinoma” OR “extrahepatic cholangiocarcinoma” OR “gallbladder cancer” OR “gallbladder neoplasms” OR “bile duct cancer” OR “bile duct neoplasms” OR “pancreatic cancer” OR “pancreatic carcinoma”):ti,ab,kw
