# Supplementary figures and images for: The advanced lung cancer inflammation index is a prognostic factor for gastrointestinal cancer patients undergoing surgery: a systematic review and meta-analysis
Source: World J Surg Oncol. 2023 Mar 6;21:81. doi: 10.1186/s12957-023-02972-4 (PMC9987069; doi:10.1186/s12957-023-02972-4)

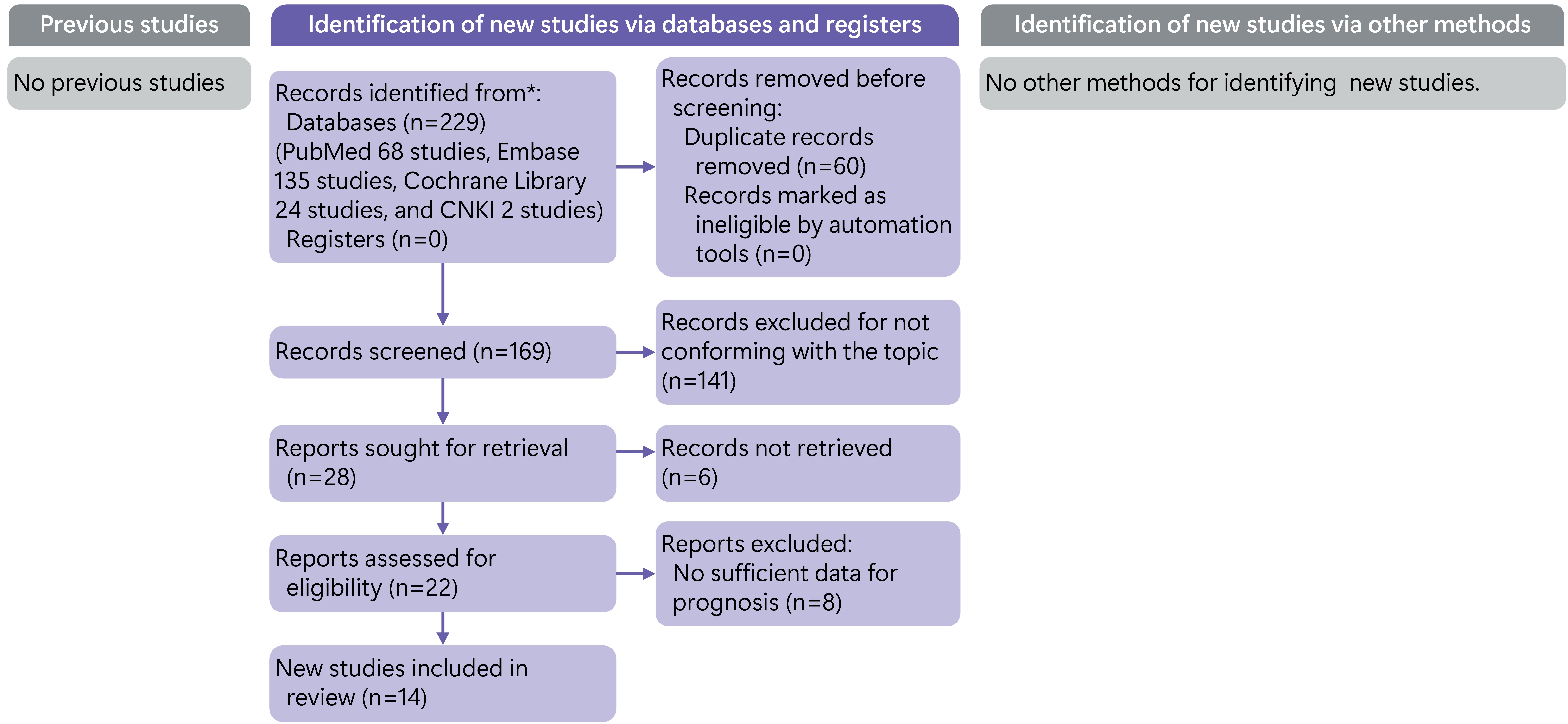

Supplement: Supplementary file 4 — Additional file 4. PRISMA flow chart. [file 12957_2023_2972_MOESM4_ESM.jpg]
