# Supplementary material for: The advanced lung cancer inflammation index is a prognostic factor for gastrointestinal cancer patients undergoing surgery: a systematic review and meta-analysis
Source: World J Surg Oncol. 2023 Mar 6;21:81. doi: 10.1186/s12957-023-02972-4 (PMC9987069; doi:10.1186/s12957-023-02972-4)
Supplement: Supplementary file 5 — Additional file 5: Table S1. Results of quality assessment using the Newcastle-Ottawa Scale for cohort studies. [file 12957_2023_2972_MOESM5_ESM.docx]

Table S1 Results of quality assessment using the Newcastle-Ottawa Scale for cohort studies.

| Study | Selection |  |  |  | Comparability | Outcome |  |  | Scores |
| --- | --- | --- | --- | --- | --- | --- | --- | --- | --- |
|  | Representativeness of exposure | Selection of the non-exposure | Ascertainment of exposure | Demonstration that outcome was not present at start | Cohorts on the basis of the design or analysis | Assessment | Long follow-up for outcomes to occur | Adequacy of follow-up |  |
| Horino T (2021) [21] | ★ | ★ | ★ | ★ | ★★ | ★ | ★ | ★ | 9 |
| Pian G (2020) [22] | **☆** | ★ | ★ | ★ | ★★ | ★ | **☆** | **☆** | 6 |
| Kusunoki K (2020) [23] | **☆** | ★ | ★ | ★ | ★★ | ★ | ★ | ★ | 8 |
| Xie HL (2020) [24] | ★ | ★ | ★ | ★ | ★★ | **☆** | ★ | ★ | 8 |
| Shinutani M (2019) [25] | **☆** | ★ | ★ | ★ | ★★ | ★ | **☆** | **☆** | 6 |
| Chen C (2022) [26] | ★ | ★ | ★ | ★ | ★★ | **☆** | ★ | ★ | 8 |
| Yin CZ (2021) [27] | ★ | ★ | ★ | ★ | ★★ | ★ | ★ | ★ | 9 |
| Zhang X (2022) [28] | ★ | ★ | ★ | ★ | ★★ | ★ | ★ | ★ | 9 |
| Deng Y (2022) [29] | **☆** | ★ | ★ | ★ | ★★ | ★ | ★ | ★ | 8 |
| Tan X (2021) [30] | **☆** | ★ | ★ | ★ | ★★ | **☆** | ★ | ★ | 7 |
| Feng JF (2014) [31] | ★ | ★ | ★ | ★ | ★★ | ★ | ★ | **☆** | 8 |
| Li Q (2022) [32] | **☆** | ★ | ★ | ★ | ★★ | **☆** | ★ | ★ | 7 |
| Wu H (2022) [33] | **☆** | ★ | ★ | ★ | ★★ | **☆** | ★ | ★ | 7 |
| Barth DA (2020) [34] | ★ | ★ | ★ | ★ | ★★ | ★ | ★ | ★ | 9 |
