# Supplementary material for: The advanced lung cancer inflammation index is a prognostic factor for gastrointestinal cancer patients undergoing surgery: a systematic review and meta-analysis
Source: World J Surg Oncol. 2023 Mar 6;21:81. doi: 10.1186/s12957-023-02972-4 (PMC9987069; doi:10.1186/s12957-023-02972-4)

Egger test

OS (Fig 2a)：

P=0.00<0.05, which suggests a significant bias.


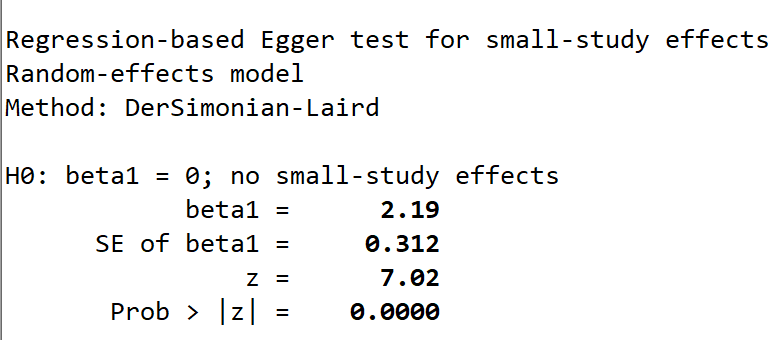


DFS (Fig 2b)：

P=0.00<0.05, which suggests a significant bias.


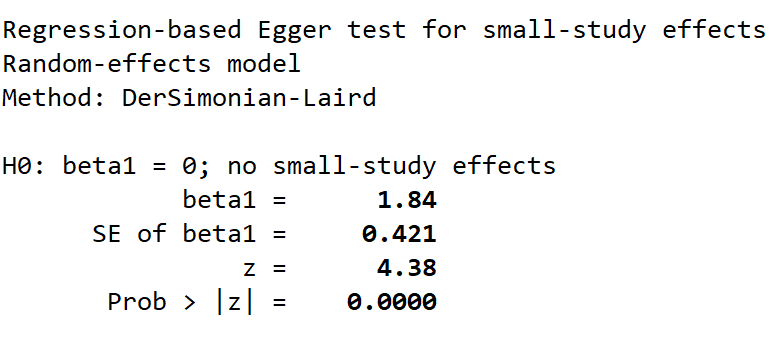


CSS (Fig 2c)：

P=0.3252>0.05, which suggests no significant publication bias.


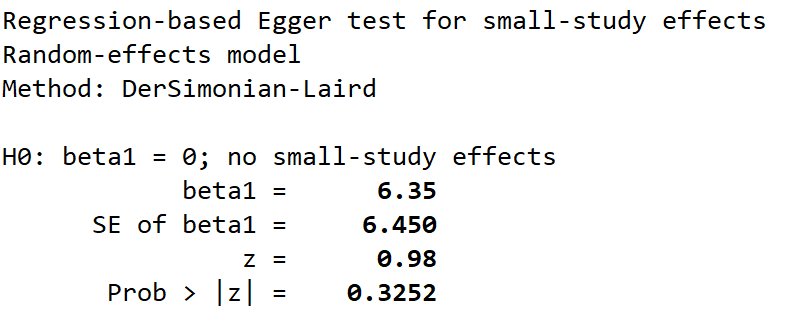

Supplement: Supplementary file 6 — Additional file 6: Table S2. [file 12957_2023_2972_MOESM6_ESM.docx]
